# G2Basy: A framework to improve the RNN language model and ease overfitting problem

**DOI:** 10.1371/journal.pone.0249820

**Published:** 2021-04-14

**Authors:** Lu Yuwen, Shuyu Chen, Xiaohan Yuan

**Affiliations:** School of Big Data & Software Engineering, ChongQing University, ChongQing, China; University of Science and Technology Beijing, CHINA

## Abstract

Recurrent neural networks are efficient ways of training language models, and various RNN networks have been proposed to improve performance. However, with the increase of network scales, the overfitting problem becomes more urgent. In this paper, we propose a framework—G2Basy—to speed up the training process and ease the overfitting problem. Instead of using predefined hyperparameters, we devise a gradient increasing and decreasing technique that changes the parameters training batch size and input dropout simultaneously by a user-defined step size. Together with a pretrained word embedding initialization procedure and the introduction of different optimizers at different learning rates, our framework speeds up the training process dramatically and improves performance compared with a benchmark model of the same scale. For the word embedding initialization, we propose the concept of “artificial features” to describe the characteristics of the obtained word embeddings. We experiment on two of the most often used corpora—the Penn Treebank and WikiText-2 datasets—and both outperform the benchmark results and show potential towards further improvement. Furthermore, our framework shows better results with the larger and more complicated WikiText-2 corpus than with the Penn Treebank. Compared with other state-of-the-art results, we achieve comparable results with network scales hundreds of times smaller and within fewer training epochs.

## Introduction

Natural language processing (NLP) is the area of artificial intelligence that concerns the automatic generation and understanding of human languages [1]. Language models are an essential part of NLP that can predict upcoming words based on a given context [2]. It can be used in a wide range of applications such as question answering, sentiment analysis, machine translation, speech recognition, etc. Language models generated by neural networks are first proposed by Xu and Rudnicky [3] in 2000, and later are further studied by Bengio and Ducharme [4]. Their work inspires more sophisticated models that show great performance on multiple NLP applications [5–8]. Some of the research objectives of language modeling drifted from training models to acquiring word embeddings ever since. After the introduction of RNNs in [9], distributed word representations have shown superior performance over traditional NLP routines, and thus becomes more received in both academic and industry. Deep learning brings new representations, architectures, and techniques for NLP researches. However, by introducing deep neural networks, we also bring its intrinsic defects to those NLP tasks, such as uninterpretable results, overfitting, and difficulty of training.

Due to the large size of the training corpus and network parameters, training word embeddings can take weeks or more. Therefore, it makes sense to speed up the training time by initializing new networks with pretrained word embeddings [6]. And the process of using pretrained initialization can go on recursively according to [10]. Here, we initialized the input word embeddings with the pretrained GloVe [11] and design our framework based on it. Unlike its preceding models, GloVe takes into consideration the overall co-occurrence statistical information of the whole training corpus. Therefore, among its obtained word vectors, the Euclidean distance reflects their semantic similarities. This prompted us to derive the idea of initializing an RNN with randomly selected pretrained word vectors. Since the space comprising pretrained word vectors is only a small subset of the whole multidimensional space it is in, we presume that initializing with randomly selected pretrained word embeddings, can outperform those initialized with random data generated from certain distribution. After all, the word vectors are obtained from hundreds of millions of trainings, making them more reliable.

To alleviate the overfitting problem and enhance the generalization ability of language models, mechanisms like tied weights [12], dropout [13], and a vast variety of optimization algorithms, such as Momentum [14], Adadelta [15], and Adam [16], have been proposed. However, these techniques do not work well on RNNs, especially on LSTM networks [17], which are designed to solve long time lag tasks. Still, multiple approaches have been used to apply dropout—one of the most successful regularization techniques—to RNNs [16–18]. Zaremba et al. [19] apply dropout only to the nonrecurrent connections. Gal et al. [20] randomly drop inputs, outputs, and recurrent connections at each time step. Song et al. [21] conduct dropout with a regular and online generated pattern to eliminate unnecessary computation and data access. In our experiments, we apply dropout only to the network input and tie the input and output vectors, as done in [12]. Then, we treat the dropout hyperparameter as a variable and change its values during the training process.

In this paper, we propose an LSTM-based framework. It uses the pretrained GloVe word embeddings to initialize its input vectors and changes optimization algorithms during training. Together with the hyperparameter updating strategies we propose, our framework offers quicker convergence in much shorter training time compared with the benchmark. We call our framework G2Basy: the **G**radient **Ba**tch **S**ize and H**y**brid optimizers framework with pretrained **G**loVe vectors. Experiments conducted on the PTB [22] and WikiText-2 [23] corpora show that our framework outperforms the benchmark model. Furthermore, compared with other state-of-the-art regularized multilayer RNN models with much larger scales, our framework still achieves close results.

## Materials and methods

As mentioned above, we use public corpora –PTB and WikiText-2—to train, evaluate and test our G2Basy framework. And it is covered in three aspects, i.e., word vector initialization, hyperparameter update strategy, and other auxiliary techniques. In this section, we focus on some hyperparameters that are usually overlooked. When estimating performance, reaching equivalent results within less training time is also one of our goals, besides smaller test perplexity.

### Experiments datasets

We conduct word-level prediction experiments on the PTB dataset, preprocessed by [22], which consists of 929k training words, 73k validation words, and 82k test words. After testing all of our training strategies on the PTB corpus, we apply them to the larger and more sophisticated WikiText-2 corpus to further test the generalization ability of our framework. The statistics for the corpora are listed in Table 1 below.

**Table 1:** Statistics information of the PTB and WikiText-2 datasets.

	Penn Treebank			WikiText-2		
	Train	Valid	Test	Train	Valid	Test
Articles	-	-	-	600	60	60
Tokens	929,590	73,761	82,431	2,088,628	217,646	245,569
Vocab size	10,000			33,278		
Oov rate	4.8%			2.6%		

Note: The out of vocabulary (OoV) rate notes what percentage of tokens have been replaced by an <unk> token. The token count includes newlines which add to the structure of the WikiText dataset.

### Initialization

From the informatics point of view, words can be treated as encoders of the entities or meanings to which they refer. Therefore, in addition to morphology information such as word form, tense, and affix [24–26], other statistical information such as the direction of word embeddings and training sentence length can be used as indicators for further analysis. If we assume that there exists one certain vector for each specific word, then compared to the whole multidimensional space, the pretrained word embeddings, therefore, belong to a much more compact subspace. Before further discussion, we define “unseen words” as those in the corpus that don’t have a counterpart in the vocabulary of the GloVe pretrained embeddings and refer to them as such from now. Then we assume that when using pretrained word embeddings, it may improve the results if we initialize the unseen words with randomly selected pretrained word vectors (or vectors pointing in the same direction). We also tried to push it further and initialize all the words with random pretrained word vectors.

When we treat the unseen words separately, there are two ways to initialize them, i.e., with data generated from a uniform distribution U(-0.1,0.1] (abbreviated as “Uniform” later) or other distributions and with vectors randomly selected from GloVe. From now on, we use R(GloVe) to represent initializing words with random GloVe vectors, R(Glove/2) to represent initializing with random GloVe vectors divided by scalar 2, and R(GloVe/4) to represent initializing with random GloVe vectors divided by scalar 4. A full initialization process is, therefore, written as GloVe+R(GloVe/2), and its two parts represent the initialization of the words that have counterparts in the pretrained embeddings and those unseen words, respectively.

### Hyperparameter updates

If we keep the model structure unchanged when training neural networks, then optimization algorithms become one of the key factors to acquiring better performance. Many optimization algorithms help adjust gradients better and faster, but SGD is still dominantly used [27, 28] and remains one of the most robust. Therefore, we choose the mini-batch version of basic SGD with simulated annealing as the benchmark to evaluate the performance of our framework.

Among all the possible hyperparameters, we focus on two sets of them. The first is the selection of optimization algorithms and the second is the training batch size and the dropout of network input. De, Goyal, and Smith et al. [29–31] have proved that changing the value of the training batch size improves model performance. Balles [32] proposed a strategy that couples the training batch size and learning rate which yields faster optimization convergence and simultaneously simplifying learning rate tuning. In this paper, we tune the training batch size and the dropout value simultaneously in an adaptive-like way since the learning rate parameter is already taken care of during the simulated annealing process in our G2Basy framework.

The benchmark training parameter settings are listed in Table 2 below. We referred to other researches [20, 23, 33, 34] that use the same dataset when setting those parameters, and then conducted a grid search to finetune and verify them. As for parameters “Layers” and “Word vector Dimension” we only choose their values as in Table 2 because they suit our computational resources, any increase would make the training time unbearable. We prefer uniform distribution for two reasons. Theoretically, it doesn’t make priori assumptions about the data distribution. And in our experiments, it shows better performance and robustness compared with other distributions such as Gaussian.

**Table 2:** The parameter settings of the benchmark training.

Parameters	Settings	Comments
RNN Types	LSTM	RNN network type
Layers	2	Layers of RNN network
Word vector Dimension	200	
Word vectors Initialization	U(-0.1,0.1)	Initialized by uniform distribution raging from -0.1 to 0.1
Parameter Clip	0.25	Clip parameters
Learning Rage	20	Initial learning rate
Decay Rate	0.25	Multiply 0.25 when annealing proceeds
Training Batch Size	25	
Evaluation Batch Size	10	
Dropout	0.20	Apply dropout to input layers
Tied Parameters	True	While training tie input and output layers

#### Optimizer algorithms

Almost every optimizer has a default setting for the learning rate at which they usually perform best. If their mechanisms of gradient updates do not counteract with each other, then the use of multiple optimization algorithms can improve training performance, similar to feed the output of one network as the input to another.

Adadelta accumulates gradients among a fixed window instead of all the gradients, and it also corrects units with Hessian Approximation. Although it is designed not to set the learning rate manually, when initialized with network parameters pretrained by SGD, better results can be obtained within fewer training epochs in a shorter time. It works even better when pretrained word embeddings are used. The pseudocode of Adadelta is depicted in Table 3 below. For more detailed information, refer to [15].

**Table 3:** The pseudocode of Adadelta optimization algorithm.

Algorithm 1 Computing Adadelta update at time t
Require: Decay rate *ρ*, Constant *ϵ*
Require: Initial parameter *x*_1_
1. Initialize accumulation variables *E*[*g*^2^]_0_ = 0, *E*[Δ*x*^2^]_0_ = 0
2. for *t* = 1: *T* do %% Loop over #of updates
3. Compute Gradient: *g*_*t*_
4. Accumulate Gradient: $E{\left[g^{2}\right]}_{t}=\rho E{\left[g^{2}\right]}_{t-1}+\left(1-\rho \right)g_{t}^{2}$
5. Compute Update: $\Delta x_{t}=-\frac{RMS{\left[\Delta x\right]}_{t-1}}{RMS{\left[g\right]}_{t}}g_{t}$
6. Accumulate Updates: $E{\left[\Delta x^{2}\right]}_{t}=\rho E{\left[\Delta x^{2}\right]}_{t-1}+\left(1-\rho \right)\Delta {x_{t}}^{2}$
7. Apply Update: *x*_*t*+1_ = *x*_*t*_ + Δ*x*_*t*_
8. end for

Note: RMS stands for Root Mean Square.

One typical way of finding a more appropriate learning rate is simulated annealing. And it usually begins with a large initial learning rate which decays during training. Furthermore, because of the intrinsic differences of parameter updating among different optimization algorithms, introducing another new optimizer does not necessarily work better. Besides SGD, the other optimizer we choose is abbreviated as ASGD [35]. It is a recursive algorithm of stochastic approximation type with the averaging of trajectories. ASGD improves the previous stochastic approximation methods so that it does not require a large amount of a priori information. It is based on the following paradoxical idea: a slow algorithm having less than optimal convergence rate must be averaged [36, 37]. And for stochastic optimization, consider the problem of searching for the minimum x* of the smooth function *ℓ*(*x*), $x\in R^{N}$. The values of the gradient *y*_*t*_ = ∇*ℓ*(*x*_*t*−1_ + *ξ*_*t*_) containing random noise *ξ*_*t*_ are available at an arbitrary point *x*^*t*−1^ of $R^{N}$. To solve this problem, the following recursive algorithm of averaging is proposed:

$$\begin{array}{c} x_{t}=x_{t-1}-\gamma_{t}\varphi \left(y_{t}\right) \end{array}$$ (1)

$$\begin{array}{c} y_{t}=\nabla \ell \left(x_{t-1}\right)+\xi_{t} \end{array}$$ (2)

$$\begin{array}{c} {\bar{x}}_{t}=\frac{1}{t}\sum_{i=0}^{t-1}x_{i}\;x_{0}\in R^{N} \end{array}$$ (3)

Under the following conditions:

Assumption 1. *ℓ*(*x*) is a twice continuously differentiable function and *lI* ≤ ∇^2^*ℓ*(*x*)≤*LI* for all *x* and some *l* > 0 and *L* > 0; and *I* is the identity matrix.
Assumption 2. (*ξ*_*t*_)_*t* ≥ 1_ is the sequence of mutually independent and identically distributed random variables and *Eξ*_*t*_ = 0.
Assumption 3. It holds that:
  $$\begin{array}{c} \left|\varphi \left(x\right)\right|\leq K_{1}\left(1+|x|\right) \end{array}$$ (4)

And four more conditions must be satisfied for the algorithm to work properly regarding the relationships among *γ*_*t*_, *ℓ*(x*) and *φ*(*x*). Refer [35] for more detailed information and the derivation procedure.

#### Batch size and dropout update

Another powerful regularization procedure is dropout [13, 18, 20, 21], but it does not work well on RNNs. Thus, when used in RNNs, it is usually applied only to the input and output layers. During the basic simulated annealing procedure, as the learning rate decreases, the model’s improvement tends to slow down and get stuck or show oscillation. As we mentioned at the beginning of this section, training batch size and dropout are tuned simultaneously. When training RNN language models, changing training batch size can not only shorten the training time but also provide input with different contexts. This offers more possible information about the corpus for our model to learn, so better results can be expected if variety is introduced to the value of batch size.

The specific steps of the gradient batch size and dropout updating algorithm are shown in Table 4. The “reset random seed” in step (Step 9) is necessary since accumulated long dependencies can harm the RNN’s generalization ability. It is also an indispensable procedure for the training initialized with pretrained word vectors to work. The essential idea of the “reset random seed” step is to add disturbance to the dropout procedure in the forward function of the RNN model. This step brings uncertainty to the dropout as to which input word vectors will make it to the model. To reset the manual seed, simply change the dropout pattern of the input vectors. One way to do this is to change the random seed of the dropout procedure.

**Table 4:** The pseudocode of the gradient batch training algorithm.

Algorithm 2 Batch size and dropout update with annealing procedure
Require: Batch size *β*, Batch size updating step size Δ*β*
Require: Dropout *ρ*, Dropout updating step size Δ*ρ*
Require: User-defined minimum batch size *min*_*β* ^1^
1. while the stopping criteria are not reached:
2. Initialize batch size and dropout: *β* = *β*_0_, *ρ* = *ρ*_0_
3. Set *best*_*loss* to store the recent best *valid*_*loss* value
4. For every training epoch, if *best*_*loss* updates:
5. reset random seed ^2^
6. *β*+ = Δ*β*, *ρ*+ = Δ*ρ*
7. else train until annealing happens:
8. if *β* ≥ *min*_*β*:
9. reset random seed
10. *β*− = Δ*β*, *ρ*− = Δ*ρ*
11. else:
12. keep *β* and *ρ*, and run the basic annealing training
13. end while

Note:

^1^.The value of this parameter is different for different corpora.

^2^.Reset random seed refers to the procedure of adding disturbance to the dropout step.

**Fig 1:**
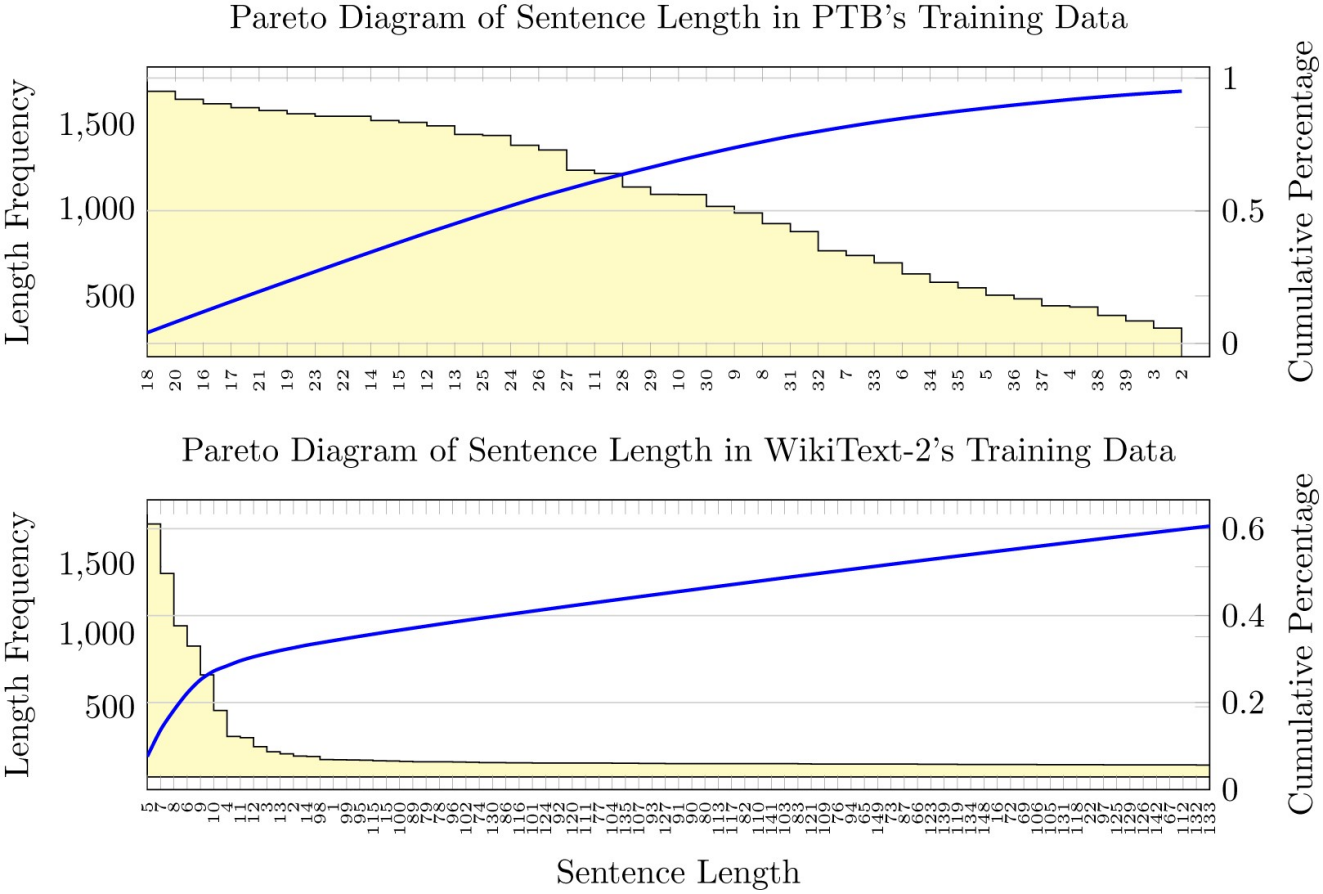
Pareto diagram of sentence length in corpora’s training data.

To use the gradient batch size training of the PTB dataset as an example, we start the training with batch size 10 and dropout 0.05 and increase them by 5 and 0.05 respectively after each training epoch until the so-far best valid loss stops updating. We then follow the training procedure of basic simulated annealing, but when annealing happens, we update not only the learning rate but also the batch size and dropout. We stop updating batch size when it decreases to 30 and keep it at that value until the training stops. The batch size value 30 here corresponds to the hyperparameter *min*_*β* in Table 4, and we found it manually because it offers better results in a grid search. As the Pareto diagram shown in Fig 1, the sentence length of training text in PTB corpus falls mostly in the range of 10 to 30. Yet the lengths distribution of the WikiText-2 corpus is far more different from the PTB, most of its sentences are much longer, and cover a larger length range.

#### Training stopping criteria

Valid loss is a traditional criterion to monitor the training conditions. If the number of training epochs is preset, usually the model with the minimum valid loss is chosen as the final result. In our situation, since we are training to find the limits of the training strategies we propose, therefore, it is not practical to preset the training epoch number or to train without considering early stopping. During early exploratory training, for each training epoch we observe both the valid loss and test loss and select a set of stopping criteria according to experimental observations and conventional standards.

Before presenting our stopping criteria, we will first introduce the parameters used. Our basic training process is simulated annealing, and annealing happens when the current valid loss becomes larger than the so-far best. In an ideal situation, the valid loss drops below the so-far best valid loss right after the annealing epoch. However, in other situations, it does not drop, thus causing another annealing to occur, i.e. annealing happens twice in a row. For situations like this, we define the parameter Continued_Anneal_Num as the number of continuously annealing epochs. Table 5 below is an example of continuously annealing when Continued_Anneal_Num equals 3.

**Table 5:** An example of Continued_Anneal_Num equals 3.

Epoch Number	Current valid PPL	Best valid PPL	Continued_Anneal_Num
i	100	100	Update best valid perplexity
i+1	105	100	1
i+2	103	100	2
i+3	101	100	3
i+4	99	99	Reset to 0
i+5	98	98	Remains 0

We set different stopping criteria for different training procedures based on the exploratory experiments conducted on the PTB dataset. During training, we set Continued_Anneal_Num as a hyperparameter and set different threshold values of it for different training procedures. For basic benchmark annealing training, if Continued_Anneal_Num reaches 5, the training procedure trains one more epoch and then takes the better valid perplexity (PPL) between the last two training epochs as the final result. For the strategy of different optimizers, we stop the training when the first annealing happens after introducing ASGD and take the epoch before annealing as the final result. The stopping criterion for gradient batch size is similar to basic annealing, just change the threshold value of Continued_Anneal_Num from 5 to 7.

#### Learning rate back-tracking with ASGD

There is another way of getting better perplexity results with ASGD, which is changing the optimizer from SGD to ASGD when the learning rate is close to 0 and increasing the learning rate. In our experiments, we set the increased learning rate back to 0.02, since in the basic simulated annealing procedure ASGD performs best at a learning rate of 0.01953125. We call this strategy Learning Rate Back-tracking. A pitfall of the back-tracking is that it requires more training time. A more specific description of this method is illustrated in Table 6 below.

**Table 6:** The pseudocode of the learning rate back-tracking.

Algorithm 3 The Procedure of Learning Rate Back-tracking
Require: Learning rate *lr*
Require: User-defined parameter $\tilde{lr}$
1. while the stopping criteria is not reached:
2. if *lr* → 0:
3. change optimizer to ASGD
4. $lr=\tilde{lr}$
5. reset random seed
6. if overfitting happens:
7. break
8. end while

Note: learning rate *lr* can be as small as 1 ⋅ 10^−8^ or less, and $\tilde{lr}$ is usually set to 0.02.

### G2Basy: A framework to combine them all

**Fig 2:**
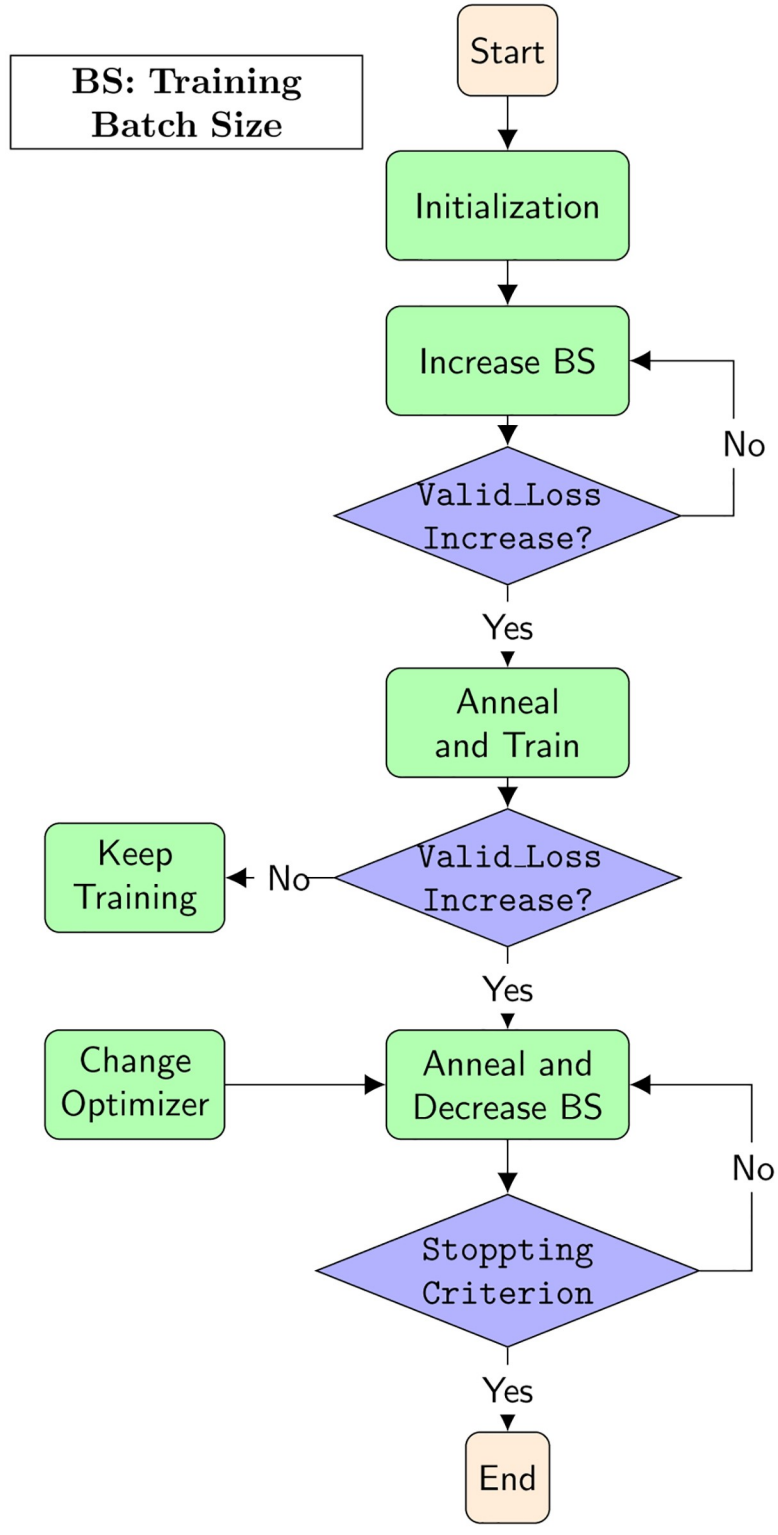
The diagram of G2Basy framework.

We call this **G**radient **Ba**tch **S**ize and H**y**brid optimizers framework with pretrained **G**loVe word embeddings initialization G2Basy. Its overall diagram is illustrated in Fig 2. For the G2Basy framework, by combining all the strategies mentioned above, the best expectation is that they all add positive effects to the final results. However, the experimental results tell us differently. The combination of gradient batch size and the ASGD optimizer does not work out with the PTB dataset as well as we expected. In addition, there is another problem that does not show up in the diagram of Fig 2, i.e., the timing of introducing the ASGD optimizer. The default setting of the learning rate parameter in ASGD is 0.01, which we considered when choosing the introduction time. There are some trade-offs between training time and the test perplexity when ASGD is brought in at different learning rates. The larger the learning rate, the quicker it reaches a local minimum and overfits which shows during the training process as higher and higher valid perplexity. In the next section, we will have a more detailed discussion of when to bring in the ASGD optimizer and how it influences the model’s performance.

## Results and discussion

There are three key factors in our G2Basy framework, i.e., initializing with pretrained word vectors, introducing different optimizers during training, and gradient updating of training batch size and dropout. They can improve results singly and offer even better results if combined. As shown in Table 7 in the next section, pretrained word vectors offers the best outcomes, followed by the gradient batch size and dropout updating procedure, which shows good performance among all different initializations. If parallel computing is not considered, training time per epoch decreases with the increase in training batch size. However, the time differences among different batch sizes are not very distinct and can be easily influenced by the state of the currently running machine. Therefore, when we talk about training time hereafter, we refer to the number of training epochs.

**Table 7:** Perplexity of different initializations and improvement strategies.

Initialization	Procedure	Valid PPL	Test PPL	Stopping Epoch	Best Test PPL	Best Test Epoch
Uniform	Annealing^1^	91.1	86.6	72	86.6	86
	Optimizer1^2^	88.6	85.0	122	85.0	122
	Optimizer2^3^	88.9	85.1	58	85.1	58
	Batch Size^4^	87.7	***83.5***	89	***83.1***	***122***
R_ GloVe/2	Annealing	89.8	85.7	68	85.6	77
	Optimizer1	87.9	84.6	105	84.6	105
	Optimizer2	87.8	84.4	62	84.4	62
	Batch Size	87.6	***83.6***	111	83.6	111
R_ GloVe/4	Annealing	90.0	86.1	59	86.1	111
	Optimizer1	88.2	85.0	114	85.0	114
	Optimizer2	88.4	84.8	59	84.8	59
	Batch Size	86.7	***83.1***	98	***82.9***	***116***
GloVe+ Uniform	Annealing	83.2	80.3	61	80.1	115
	Optimizer1	80.9	78.8	129	78.6	142
	Optimizer2	80.6	***78.4***	72	78.4	72
	Batch Size	81.9	78.7	86	***78.4***	***143***
GloVe+ R(GloVe/2)	Annealing	83.8	80.6	54	80.4	60
	Optimizer1	82.0	79.2	98	79.1	104
	Optimizer2	81.9	79.1	57	79.1	57
	Batch Size	82.5	***78.6***	98	***78.2***	***136***

Note: In procedure column, there are four combinations tested for every initialization:

^1^. Annealing: basic simulated annealing procedure

^2^. Optimizer1: introducing optimizer ASGD when learning rate is 0.01953125

^3^. Optimizer2: introducing optimizer ASGD when learning rate is 0.078125

^4^. Batch Size: gradient batch size training strategy

### Initialization

As mentioned earlier, we refer to those words that do not have counterparts in the pretrained GloVe embeddings as unseen words. We use R(GloVe) to represent the procedure of initializing words with random GloVe vectors, and R(GloVe/2) to represent initializing with random GloVe vectors divided by scalar 2, and R(GloVe/4) to represent initializing with random GloVe vectors divided by scalar 4. We discard R(GloVe) initialization due to its poor performance in the experiments. A possible theoretical reason for this is that the pretrained word embeddings are rather large already, thus, the model skips the process of early parameter growth, missing many possible search areas in the solution space.

In Table 7 we list the PTB’s valid and test perplexity results for different initialization and training strategies. The last three columns need some extra explanation. To evaluate the performance of our proposed strategies, we monitor the test perplexity of each training epoch and then design precise stopping criteria (see those criteria in the section “Training Stopping Criteria” on Page 7) for all the training procedures to avoid useless training and ease overfitting. The “Valid PPL” and “Test PPL” in columns 3 and 4 are reached at epoch numbers listed in column 5, “Stopping Epoch”. Those are the results we get when our stopping criteria are satisfied. The last two columns list the best test PPL and the training epoch where it is obtained, which happens after the stopping epoch. During training, after the stopping criteria are satisfied, most of the time the training results show oscillation but the trend is still pointing in the direction of better results. Although the “Best Test PPL” is better than “Test PPL” as its name indicates, usually the improvements are not proportional to the training time they consume. The oscillation situation makes searching much more unpredictable than normal model training.

**Fig 3:**
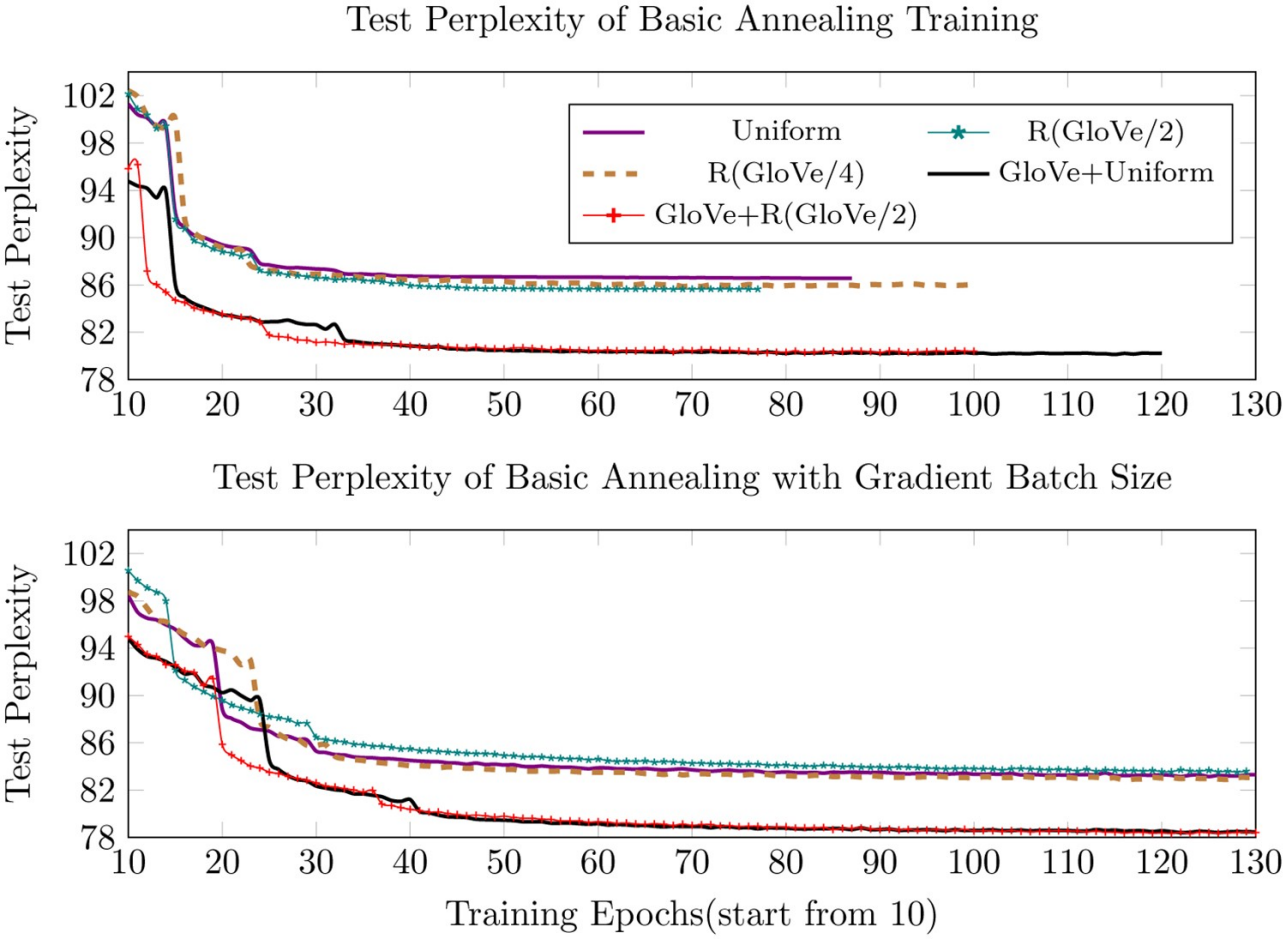
Test perplexity of basic annealing and gradient batch size.

**Fig 4:**
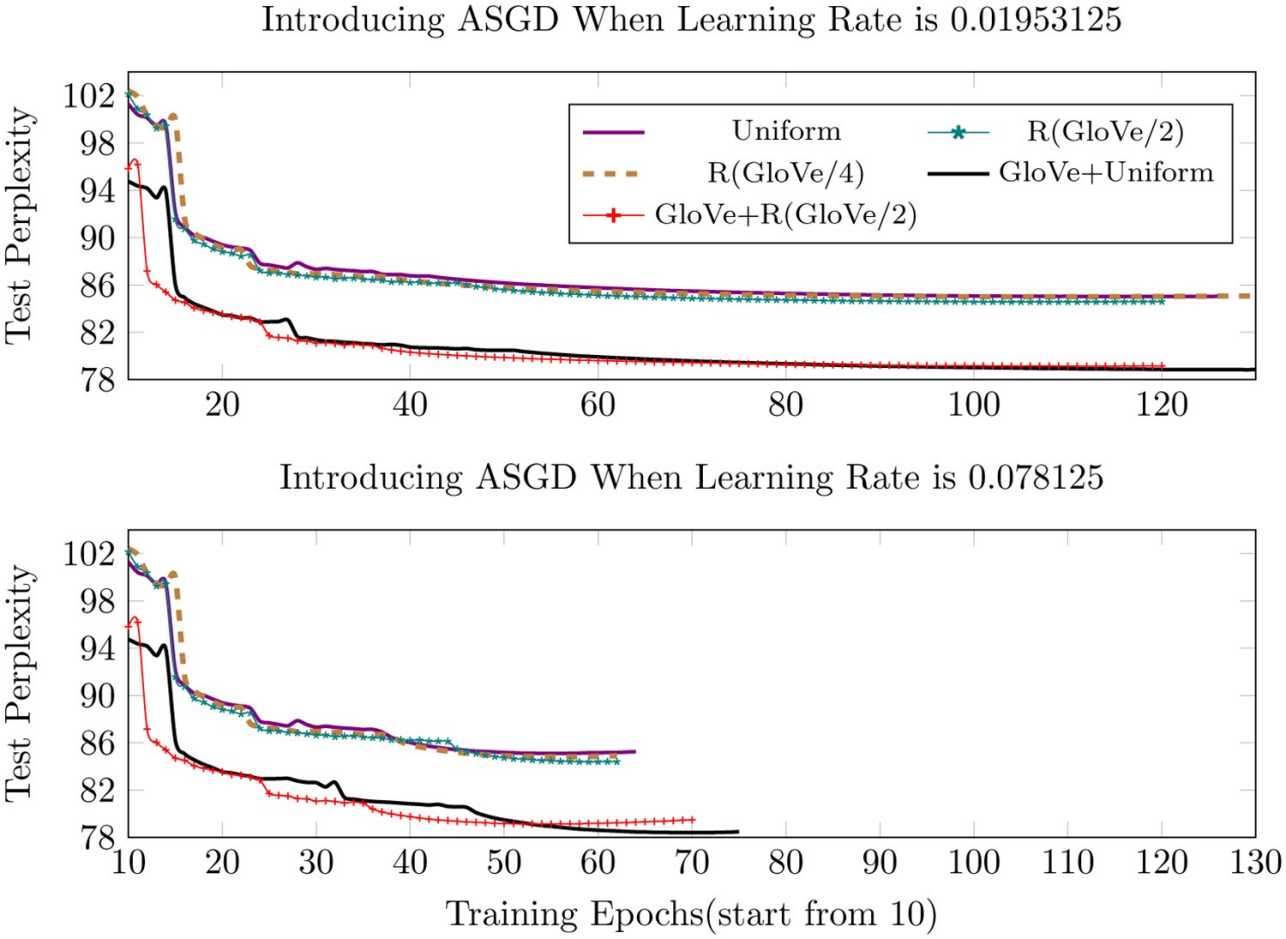
Test perplexity of introducing ASGD at different learning rates.

As shown in Figs 3 and 4 below, the pretrained GloVe embeddings make the most contribution to performance improvement. The GloVe+Uniform initialization slightly outperforms GloVe+R(GloVe/2), although we expected otherwise. However, as seen in the figures GloVe+R(GloVe/2) converges more quickly and the results become more and more similar as training proceeds. During the training of the PTB dataset, when initializing all the words with randomly selected GloVe embeddings or vectors which point in the same direction, the model converges quickly in the first few epochs, but soon reaches a not-so-satisfactory point and then either gets stuck or shows overfitting. Therefore, we only replace the words that have counterparts in the pretrained GloVe with randomly selected GloVe vectors and the others retain the random data generated from the predefined specific uniform distribution. In contrast, we initialize all the words in the WikiText-2 corpus with random GloVe vectors and it outperforms the benchmark uniform distribution initialization as we expected earlier, indicating that the random GloVe initialization works better with larger and more complicated training corpus.

The gradient batch size and dropout strategy explained in the section “Batch size and dropout update” on Page 5 improves the performance of all five initialization strategies and delays the occurrence of overfitting. The R(GloVe/4) results outperform R(GloVe/2), while with the basic simulated annealing training the situation is the opposite.

We define the above mentioned random GloVe initialization as “artificial features”. This describes the phenomenon that word vectors obtained from training, whether matching with the words or not, differ from those generated from random distributions (albeit pseudorandom). Compared with training based on characters, morphology, or so on, this broader abstraction improves the final results to a certain degree. If we can initialize the words with the pretrained word vectors of words that relate to them (which can include their synonyms, antonyms, or even words containing overlapping letters), it is foreseeable that the results are more likely to outperform those initialized by randomly distributed data. This describes a transitional state between disorder (completely random initialization) and order (perfectly matched pretrained word vectors), and the often used character-, prefix- or suffix-based methods can also be included in this framework. In specific applications, appropriate initialization techniques can be found somewhere between disorder and order according to the specific problem we are going to solve to maximize the reuse of previously trained word embeddings and improve the training results.

### Hyperparameter updates

In addition to basic SGD, we use two more optimization algorithms, i.e., Adadelta and ASGD. The effect of Adadelta is not so obvious since we only use it in between SGD and ASGD. The ASGD optimizer, however, speeds up the training time tremendously, while still offering equivalent or better results. When setting the upper limit of the batch size parameter, we referred to the statistical information of the corpus’s average sentence length, which we discussed earlier in section “Batch size and dropout” on Page 5, as well as actual exploratory training performance.

The combined strategy of changing optimizer and gradient batch size is not shown in the overall result summary in Table 7, because it does not apply to all of the initialization situations. In the R(GloVe/2) initialization, the combined strategy gets a test PPL of 83.5 at training epoch 57 when changing the optimizer from SGD to ASGD at the learning rate 0.078125. However, when using gradient batch size only, we get a result of 83.6 at training epoch 111. The ASGD optimizer almost halves the training time when reaching an equivalent test PPL. We get a similar result with GloVe+Uniform initialization—the test PPL is 78.7 at epoch 69, while the solo batch size procedure achieves the same at training epoch 87, yet its final result is 0.3 smaller than that of the combined strategy. In other words, if not very sensitive to the result, the combination of gradient batch size and ASGD optimizer can shorten the training time significantly.

We try increasing the learning rate at various points during training to see if the searching path will deviate to a better local optimum, but it does not work out. Then, we introduce a second variable—optimizer—and wait until the learning rate is very close to 0 (less than 10^−8^). We then set the learning rate to 0.02 and change the optimizer from SGD to ASGD. The training starts to converge slowly for dozens of epochs and we get the best test PPL result, 77.9, among all the trainings. Table 8 shows an overview of our learning rate back-tracking technique. There are only 3 different results instead of 5 because in the other 2 initializations the training overfits before the learning rate is small enough for us to conduct back-tracking. We change the optimizer at epoch number 100 deliberately, though this is likely not optimal. Epoch 100 was chosen as a byproduct of our exploratory training. Therefore, further research is needed to explore the back-tracking procedure with hybrid optimizers.

**Table 8:** The results of the back-tracking procedure with hybrid optimizers.

Initialization	Optimizer Changing Epoch Number	valid PPL	Obtained Epoch Number
Uniform	100	84.8	168
GloVe+Uniform	100	77.9	183
GloVe+R(GloVe/2)	100	78.1	166

The gradient training batch size strategy is a very robust procedure. It introduces two more hyperparameters, yet still reduces the search space greatly by defining the search rules and search step sizes. This strategy converges quickly within the first few training epochs and shows training potential when proper optimizers take over from SGD.

There is an extra latent hyperparameter in our framework, besides the abovementioned settings of learning rate, dropout, and training batch size—the timing of introducing different optimizers. Adadelta is introduced when the learning rate reaches 1.25 (the closest to Adadelta’s default parameter settings among all the values that occur during the simulated annealing procedure). Although it does not improve the result obviously, it delays the annealing procedure, thus ensuring that the model is trained at a rather high learning rate. Therefore, ASGD is the optimizer that makes the most difference and it works out best at a learning rate of 0.01953125 (referred to as *lr*1) and 0.078125 (referred to as *lr*2). We did not choose these learning rates for any particular reason. They are only two values among those we get when start the learning rate from 20 and divide it by 4 whenever annealing proceeds. ASGD still works if we introduce it at a learning rate of 0.3125, but the training soon begins to overfit after a few epochs. From Fig 4 above, we can see that changing the optimizer to ASGD at *lr*2 almost cuts the training time in half and the results are equivalent and even better for certain initializations. For the three randomly initialized trainings, although their lines in the figure are closely entwined, if examined closely the initialization of randomly selected GloVe vectors [both R(GloVe/2) and R(GloVe/4)] outperforms uniform distribution initialization, which partially verifies our assumption about using pretrained word vectors to narrow the search space and improve the final results.

**Fig 5:**
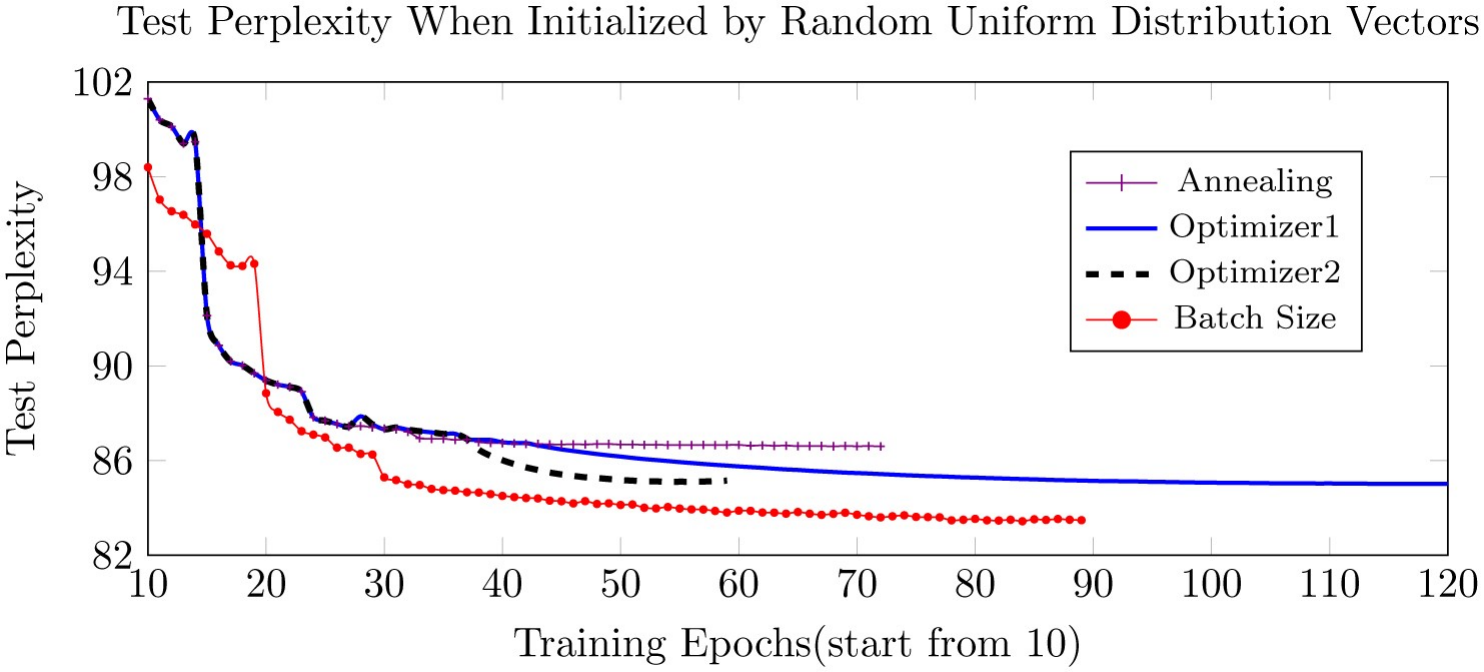
Test perplexity when initialized by uniform distributed vectors.

**Fig 6:**
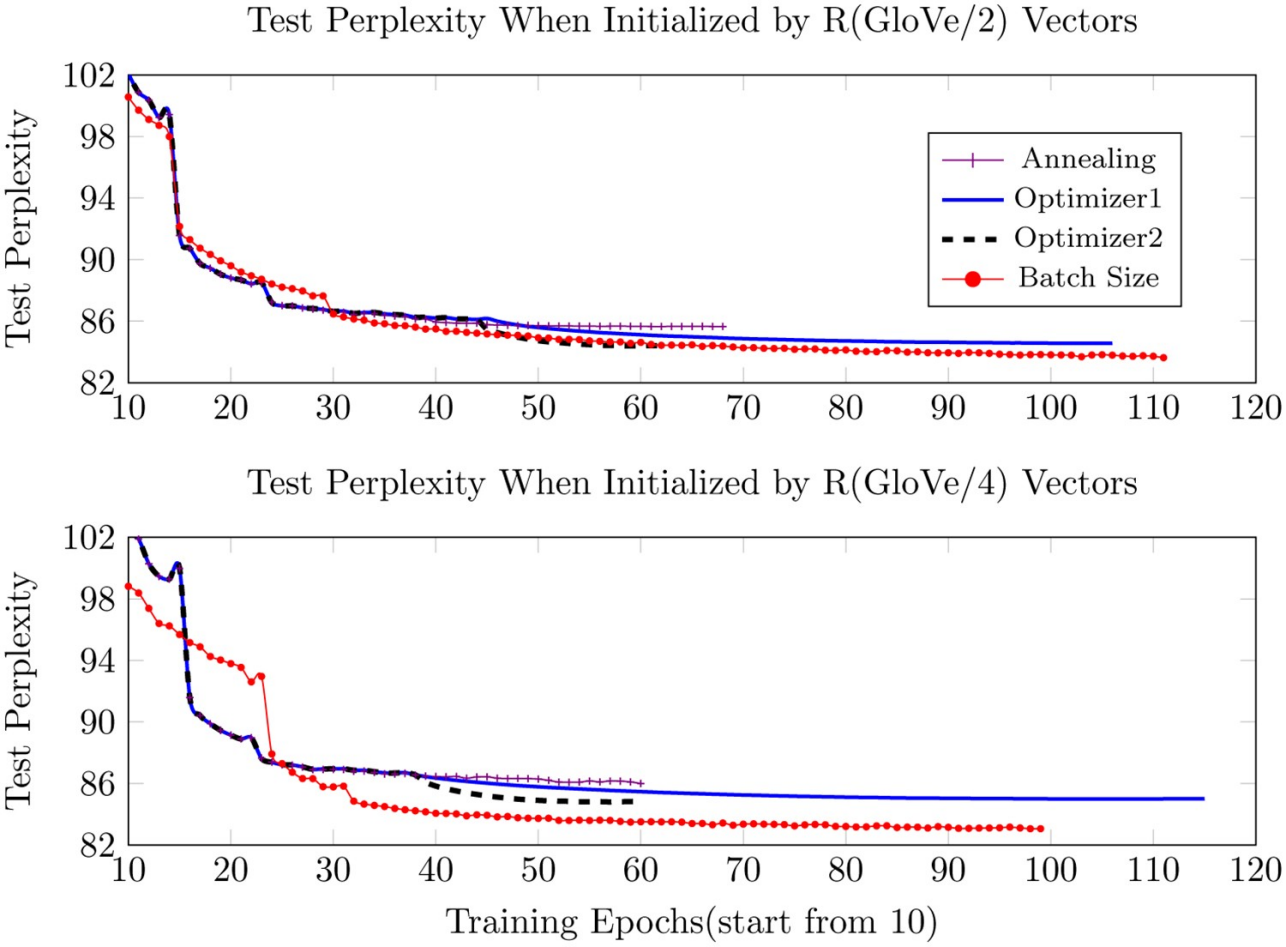
Test perplexity when initialized by random GloVe vectors.

**Fig 7:**
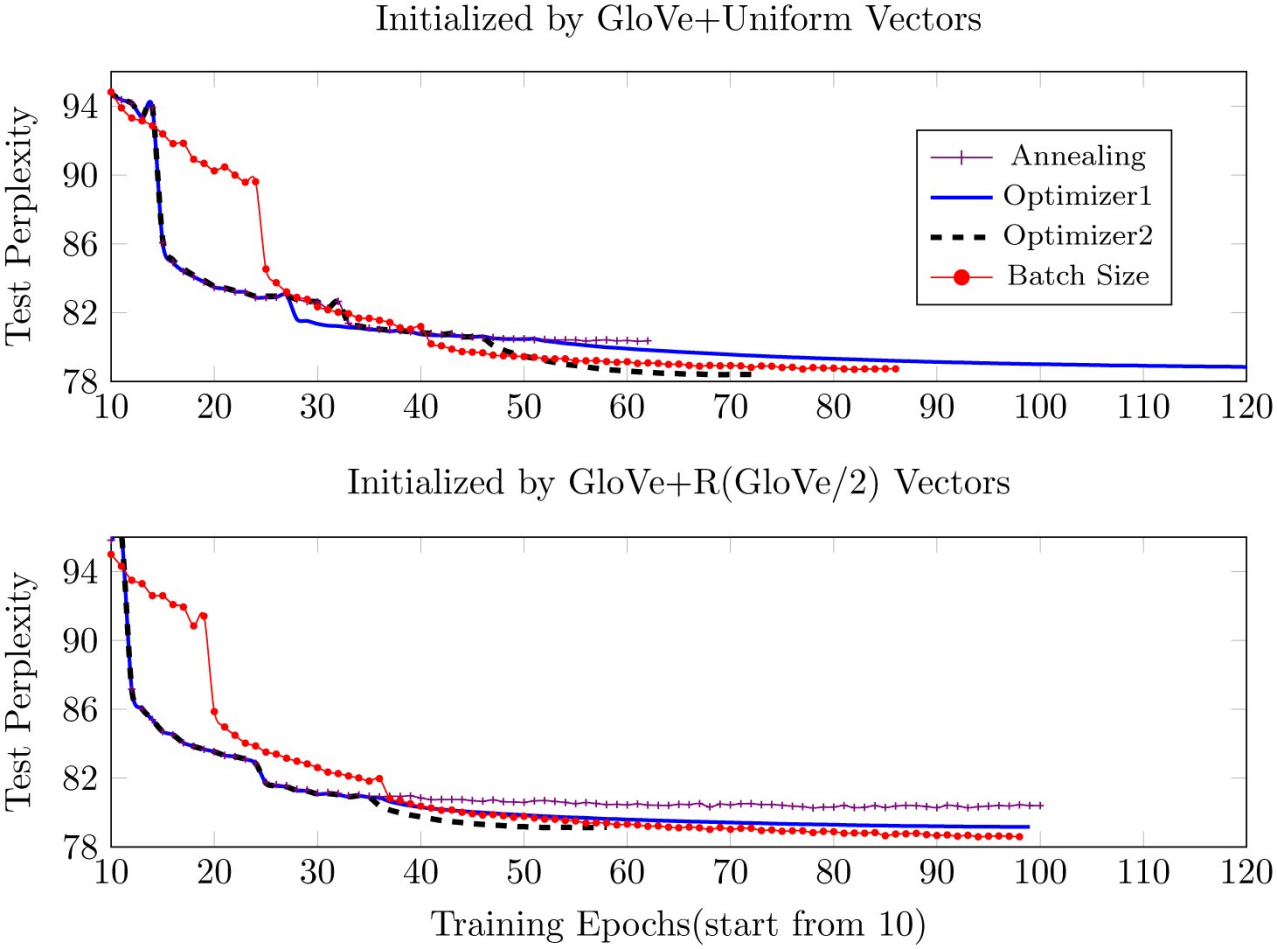
Test perplexity when initialized by precise GloVe vectors.

Figs 5–7 below show the results of different training strategies under the same distribution initialization. Annotations ‘Optimizer1’ and ‘Optimizer2’ correspond to introducing ASGD at the learning rates of 0.01953125 and 0.078125, respectively. The gradient batch size procedure provides the best results for most of the initializations, yet introducing ASGD from a rather early training stage, as done in ‘Optimizer2’, trains much faster than the other strategies and still provides the best results for precise GloVe initializations and second-best for other initializations.

### Results on PTB and WikiText-2 datasets

When network scale is considered in language modeling, there are usually 3 different model sizes, i.e., small ones with 200 units in each layer, medium ones with 650 units in each layer, and large ones with 1500 units in each layer. The number of layers is set to 2 most of the time, so we use 2 layers as well. All our models are small due to the insufficiency of computational resources.

Although there exists a gap between our test perplexity and the test perplexity obtained from state-of-the-art models, we still make a comparison with some of them [20, 23, 33, 34] and also with one of the smaller-scale models that is approximately the same size as ours [38]. The comparisons are listed in Tables 9 and 10 below.

**Table 9:** Word-level valid and test perplexity on PTB.

Model	Params	Valid PPL	Test PPL
Pointer Sentinel-LSTM(medium)(Merity et al.,2016)	21M	72.4	70.9
AWD-LSTM-MoS(Yang et al., 2018)	22M	56.54	54.44
Prune and Quantization Models(Marcin et al., 2019)	-	-	57.74
LSTM cbow WSJ (512 hidden layer) (Verwimp et al., 2020)	-	99.1	95.7
Ours-LSTM Baseline	0.64M	91.1	86.6
Ours-G2Basy Framework	0.64M	82.1	78.2

**Table 10:** Word-level valid and test perplexity on WikiText-2.

Model	Params	Valid PPL	Test PPL
Pointer Sentinel-LSTM(Merity et al.,2016)	21M	91.5	87
AWD-LSTM (Merity et al., 2017)	33M	68.6	65.8
AWD-LSTM-MoS(Yang et al., 2018)	35M	63.88	61.45
Prune and Quantization Models(Marcin et al., 2019)	-	-	86.49
Wiki-2 (512 hidden layer)(Verwimp et al., 2020)	-	119.5	111.8
Ours-LSTM Baseline	0.64M	105.9	99.9
Ours-G2Basy Framework	0.64M	96.6	90.4

## Conclusion

We propose a framework—G2Basy—that combines different initializations, multiple optimizers, and the gradient training batch size strategy, which aims to ease the overfitting problem to get better results and accelerate the training procedure. Our framework offers sufficient perplexity results using a more compact model and within fewer training epochs than conventional techniques. It also provides practical guidance for RNN language modeling training under limited computing resources. Our research offers a new perspective to look into the initialization of word embeddings. We propose the concept of “artificial features” which describes the training-developed characteristics from treating the multidimensional space consisting of all the word embeddings as a whole and taking every word embedding involved as a cross-section of the word vector space. Therefore, when dealing with problems such as polysemous words, the word vectors, and those of their contexts’, can be subjected to basic vector operations. In this case, the vector of the word is not fixed and it varies with the context of use.

If a distributed environment is available, more training optimizers and techniques can be explored to test and find more optimal solutions. In the meanwhile, the ASGD optimizer is a robust one with rather low complexity which can be generalized to other applications easily. Additionally, the strategy of combining training batch size and dropout performs well in reducing training time. Besides, the concept of “artificial features” we propose provides better outcomes and has the potential to be further studied. To sum up, the framework we propose is an efficient and robust one with easy generalization and it can offer superior results as well as faster convergence compared with the basic simulated annealing procedure.

## References

[1] Hirschberg J, Ballard BW, Hindle D. Natural Language Processing. AT&T Tech J. 1988;67(1):41–57.

[2] Cavalieri DC, Palazuelos-Cagigas SE, Bastos-Filho TF, Sarcinelli-Filho M. Combination of language models for word prediction: An exponential approach. IEEE Trans Audio Speech Lang Process. 2016;24(9):1481–1494. 10.1109/TASLP.2016.2547743

[3] Xu W, Rudnicky A. Can Artificial Neural Networks Learn Language Models? In: Proceedings of International Conference on Speech and Language Processing. Beijing, China: Speech Communication Press; 2000. p. 202–205.

[4] Bengio Y, Ducharme R, Vincent P, Janvin C. A neural probabilistic language model. J Mach Learn Res. 2003;3(6):1137–1155.

[5] Mnih A, Hinton G. A scalable hierarchical distributed language model. In: Proceedings of the 21st International Conference on Neural Information Processing Systems. NIPS’08. Red Hook, NY, USA: Curran Associates Inc.; 2008. p. 1081–1088.

[6] Collobert R, Weston J, Bottou L, Karlen M, Kavukcuoglu K, Kuksa P. Natural language processing (almost) from scratch. J Mach Learn Res. 2011;12:2493–2537.

[7] Grave E, Joulin A, Usunier N. Improving neural language models with a continuous cache. CoRR. 2016;abs/1612.04426.

[8] Shuang K, Li R, Gu M, Loo J, Su S. Major-Minor long short-term memory for word-level language model. IEEE Trans Neural Netw Learn Syst. 2019; p. 1–15. 31825875 10.1109/TNNLS.2019.2947563

[9] Mikolov T, Chen K, Corrado G, Dean J. Efficient estimation of word representations in vector space. In: 1st International Conference on Learning Representations, ICLR 2013—Workshop Track Proceedings. Scottsdale, AZ, United states; 2013.

[10] Bengio Y, Louradour J, Collobert R, Weston J. Curriculum learning. In: Journal of the American Podiatry Association. vol. 60; 2009. p. 6.

[11] Pennington J, Socher R, Manning CD. GloVe: global vectors for word representation. In: Empirical Methods in Natural Language Processing (EMNLP); 2014. p. 1532–1543.

[12] Inan H, Khosravi K, Socher R. Tying word vectors and word classifiers: A loss framework for language modeling. CoRR. 2016;abs/1611.01462.

[13] Srivastava N, Hinton G, Krizhevsky A, Sutskever I, Salakhutdinov R. Dropout: A simple way to prevent neural networks from overfitting. J Mach Learn Res. 2014;15:1929–1958.

[14] Polyak B. Some methods of speeding up the convergence of iteration methods. Ussr Computational Mathematics and Mathematical Physics. 1964;4:1–17. 10.1016/0041-5553(64)90137-5

[15] Zeiler MD. ADADELTA: An adaptive learning rate method. CoRR. 2012;abs/1212.5701.

[16] Kingma D, Ba J. Adam: A method for stochastic optimization. International Conference on Learning Representations. 2014;.

[17] Hochreiter S, Schmidhuber J. Long short-term memory. Neural Comput. 1997;9:1735–80. 10.1162/neco.1997.9.8.1735 9377276

[18] Billa J. Dropout approaches for LSTM based speech recognition systems. In: 2018 IEEE International Conference on Acoustics, Speech and Signal Processing (ICASSP); 2018. p. 5879–5883.

[19] Zaremba W, Sutskever I, Vinyals O. Recurrent neural network regularization. CoRR. 2014;abs/1409.2329.

[20] Gal Y, Ghahramani Z. A theoretically grounded application of dropout in recurrent neural Networks. In: Lee DD, Sugiyama M, von Luxburg U, Guyon I, Garnett R, editors. Advances in Neural Information Processing Systems 29: Annual Conference on Neural Information Processing Systems 2016, December 5-10, 2016, Barcelona, Spain; 2016. p. 1019–1027.

[21] Song Z, Wang R, Ru D, Peng Z, Huang H, Zhao H, et al. Approximate random dropout for DNN training acceleration in GPGPU. In: 2019 Design, Automation Test in Europe Conference Exhibition (DATE); 2019. p. 108–113.

[22] Mikolov T, Karafiát M, Burget L, Cernocký J, Khudanpur S. Recurrent neural network based language model. In: Proceedings of the 11th Annual Conference of the International Speech Communication Association, INTERSPEECH 2010. vol. 2; 2010. p. 1045–1048.

[23] Merity S, Xiong C, Bradbury J, Socher R. Pointer sentinel mixture models. CoRR. 2016;abs/1609.07843.

[24] Shi Z, Shi M, Li C. The Prediction of Character Based on Recurrent Neural Network Language Model. In: Zhu, G and Yao, S and Cui, X and Xu, S, editor. 2017 16TH IEEE/ACIS INTERNATIONAL CONFERENCE ON COMPUTER AND INFORMATION SCIENCE (ICIS 2017). Inst Elect & Elect Engineers; IEEE Comp Soc; Int Assoc Comp & Informat Sci; Wuhan Univ, Int Sch Software. 345 E 47TH ST, NEW YORK, NY 10017 USA: IEEE; 2017. p. 613–616.

[25] Adel H, Asgari E, Schuetze H. Overview of Character-Based Models for Natural Language Processing. In: Gelbukh, A, editor. COMPUTATIONAL LINGUISTICS AND INTELLIGENT TEXT PROCESSING (CICLING 2017), PT I. vol. 10761 of Lecture Notes in Computer Science. PICASSOPLATZ 4, BASEL, CH-4052, SWITZERLAND: SPRINGER NATURE SWITZERLAND AG; 2018. p. 3–16.

[26] Xiong Z, Qin K, Yang H, Luo G. Learning Chinese word representation better by cascade morphologicaln-gram. Neural Comput Appl;. 10.1007/s00521-020-05198-7

[27] Qian N. On the momentum term in gradient descent learning algorithms. Neural Netw. 1999;12(1):145–151. 10.1016/S0893-6080(98)00116-6 12662723

[28] Cui G, Guo J, Fan Y, Lan Y, Cheng X. Trend-Smooth: Accelerate Asynchronous SGD by Smoothing Parameters Using Parameter Trends. IEEE Access. 2019;7:156848–156859. 10.1109/ACCESS.2019.2949611

[29] De S, Yadav AK, Jacobs DW, Goldstein T. Big Batch SGD: Automated Inference using Adaptive Batch Sizes. CoRR. 2016;abs/1610.05792.

[30] Goyal P, Dollar P, Girshick R, Noordhuis P, Wesolowski L, Kyrola A, et al. Accurate large minibatch SGD: training ImageNet in 1 hour. 2017;.

[31] Smith SL, Kindermans P, Le QV. Don’t decay the learning rate, increase the batch size. CoRR. 2017;abs/1711.00489.

[32] Balles L, Romero J, Hennig P. Coupling Adaptive Batch Sizes with Learning Rates. CoRR. 2016;abs/1612.05086.

[33] Yang Z, Dai Z, Salakhutdinov R, Cohen WW. Breaking the softmax bottleneck: A High-Rank RNN Language Model. CoRR. 2017;abs/1711.03953.

[34] Pietron M, Karwatowski M, Wielgosz M, Duda J. Fast compression and optimization of deep learning models for natural language processing. In: 7th International Symposium on Computing and Networking Workshops; 2019. p. 162–168.

[35] Polyak B, Juditsky A. Acceleration of stochastic approximation by averaging. SIAM J Control Optim. 1992;30:838–855. 10.1137/0330046

[36] Polyak B. New stochastic approximation type procedures. Avtomatica i Telemekhanika. 1990;7:98–107.

[37] Ruppert D. Efficient estimations from a slowly convergent Robbins-Monro process. 1988;.

[38] Verwimp L, Van hamme H, Wambacq P. State gradients for analyzing memory in LSTM language models. Comput Speech Lang. 2020;61:101034. 10.1016/j.csl.2019.101034

